# An analytic, efficient and optimal readout algorithm for compact interferometers based on deep frequency modulation

**DOI:** 10.1038/s41598-024-70392-9

**Published:** 2024-09-23

**Authors:** Tobias Eckhardt, Oliver Gerberding

**Affiliations:** Institut für Experimentalphysik, Universität Hamburg, 22761 Hamburg, Germany

**Keywords:** Techniques and instrumentation, Mathematics and computing

## Abstract

Compact laser interferometers with large dynamic range are one of the core emerging tools to improve low frequency performance in gravitational wave detectors by providing local displacement sensing with sub 1 $\text {pm Hz}^{-0.5}$ precision. Strong sinusoidal frequency modulations are used in such laser interferometers to create heterodyne-like photodetector signals from which the phase and other parameters, such as the absolute distance, can be extracted. The nested sinusoidal function in such signals is a challenge for the real-time parameter estimation in low-noise applications. In this article, we present an algorithm to calculate exact signal parameters in a non-iterative way from such interferometric signals. The algorithm makes use of a recurrence relation between Bessel functions to enable a direct extraction of modulation parameters from the signal. Additionally, the algorithm is capable of dealing with high phase dynamics where the Doppler-shift of the signal becomes relevant and can limit the range and precision of the parameter estimation, if not accounted for. Simulations show that the algorithm is computationally efficient, can be well parallelised and the phase estimation is close to optimal precision given by the Cramer–Rao lower bound of the signal parameters.

## Introduction

Displacement sensing is a core metrological task that can be well addressed using laser interferometers. Compact interferometers that operate over more than a single fringe of the laser wavelength and measure in the mHz–Hz–kHz regime with sub 1 $\text {pm Hz}^{-0.5}$ precision are studied for fundamental and applied research. This prominently includes the displacement readout of ultra-precise inertial sensors for space-based geodesy missions and gravitational wave experiments^1–4^ and local inertial and displacement sensing in current and future ground-based gravitational wave detectors like LIGO, Virgo, KAGRA and the Einstein Telescope^5–8^. E.g. in LIGO, dozens of very compact and precise displacement sensors are required to isolate the mirrors from the ground-motion. More precise sensors that probe the 6 degrees of freedom can improve the mirror alignment, better suppress the mirror motion at mechanical suspension resonances and improve the active seismic isolation. Higher readout precision will generally result in lower mirror control noise, which is currently dominating detectors at low frequencies. Having compact sensors enables their deployment within the complex suspension systems^9^.

A class of interferometers studied for such applications use a scheme that we refer to here as Deep-Frequency Modulation Interferometry (DFMI)^10^. In DFMI a laser is strongly modulated in frequency and sent into several unequal arm-length interferometers, achieving both compact form and high precision displacement readout^1,11^. The interferometric signals in DFMI contain a nested sinusoidal function, which *hides* the desired signal parameters. To provide a linear estimate of these parameters the first realisations of DFMI have relied on an iterative fit algorithm that was developed by Heinzel et al. for a related technique called Deep-Phase Modulation Interferometry (DPMI)^12^. DPMI can be described as a modern variant of techniques like Phase Shifting Interferometry^13^. It uses a strong phase modulator in one arm of an interferometer (or multiple interferometers) to generate heterodyne like signals which are effectively the same as for DFMI. The iterative fit used for DFMI and DPMI, as well as other readout algorithms studied for DFMI^14^ and similar techniques^13,15–17^, use either approximations (which limit the precision) or computationally expensive, iterative fitting routines, giving them a disadvantage for a high precision test mass readout or the realisation of many channels and sensors. This paper presents an analytic algorithm to realise this parameter estimation in an efficient and optimal way for both DFMI and DPMI.

The algorithm we present aims to improve upon the readout by making use of a recurrence relation between the (non-linear) Bessel functions that appear when describing the signal in Fourier domain. It allows us to calculate all appearing parameters analytically without the need for approximations while achieving close to optimal precision (given by the Cramer–Rao lower bound of the estimates^18^). This is necessary to achieve typical precision levels around or below $100 \, {\text {fm}/\sqrt{\text {Hz}}}$ in displacement^1^, corresponding to a phase noise of $\approx 4 \cdot 10^{-7} \, \text {rad}/\sqrt{\text {Hz}}$ for a laser wavelength of 1550nm. Additionally, we extend this algorithm to operate even with larger linear dynamics and we provide simulation results to verify and quantify the algorithms performance.

**Table 1:** Nomenclature of the signal parameters.

$A$	Amplitude	$P_0$	Mean laser power
$B$	Offset	$\omega _0$	Mean laser frequency
$m$	Modulation index	$\Delta \omega$	Modulation depth
$\psi$	Modulation phase	$f_S$	Sampling frequency
$\varphi$	(Global) phase	$f_R$	Readout frequency
$\omega _m$	Modulation frequency	$t$	Time
$\Delta L$	Arm-length difference	$\tau$	Propagation-time
			(of the light)

**Fig. 1:**
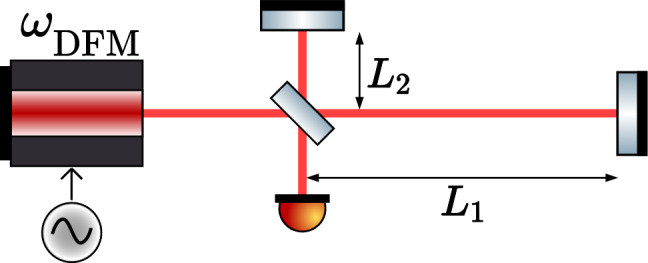
Sketch of a DFMI setup with laser frequency $\omega _{\text {DFM}} = \omega _0 + \Delta \omega \sin (\omega _m t + \psi )$ and its unequal arm-lengths. The setup measures the arm-length difference $\Delta L:= L_2 - L_1$ once encoded in the phase $\varphi$ (referred to as microscopic distance)and once in the modulation index *m* macroscopic arm-length difference $\Delta L := L_2 - L_1$.

## The signal

Figure 1 shows the core topology of DFMI. The general form of the measured signal is given by

1 $$\begin{aligned} s(t) =: B + A \cos \left( m \cdot \sin \left( \omega _m t + \psi \right) + \varphi \right) . \end{aligned}$$

For the coefficients we use the nomenclature used by^10^ as written in Table 1.

The main parameter of interest is the phase $\varphi$, which encodes the microscopic displacement of the test mass, as well as other relevant information, like relative beam tilts if a quadrant photodiode is used for differential wavefront sensing^19–21^.

Using the Jacobi-Anger identity, we write the Fourier series of the signal (1) as

2 $$\begin{aligned} s(t) = B + A \sum _{n \in {\mathbb {Z}}} J_n(m) \ \cos \bigg ( n (\omega _m t + \psi ) + \varphi \bigg ) \end{aligned}$$

with $J_n(m)$ as Bessel function of the first kind. Using the symmetry of the Bessel functions $J_{-n}(x) = (-1)^n J_n(x) \ \text {for} \ n \in {\mathbb {Z}}$ we can also rewrite the series with a summation index $n \in {\mathbb {N}}$ as

3 $$\begin{aligned} s(t) =&B + A \, J_0(m) \, \cos \varphi + 2 A \sum _{n=1}^{\infty } \Bigg [ J_{2n}(m) \cdot \cos \varphi \cdot \cos \bigg (2n(\omega _m t + \psi )\bigg ) - J_{2n-1}(m) \cdot \sin \varphi \cdot \sin \bigg ((2n-1)(\omega _m t + \psi )\bigg ) \Bigg ] \end{aligned}$$

**Fig. 2:**
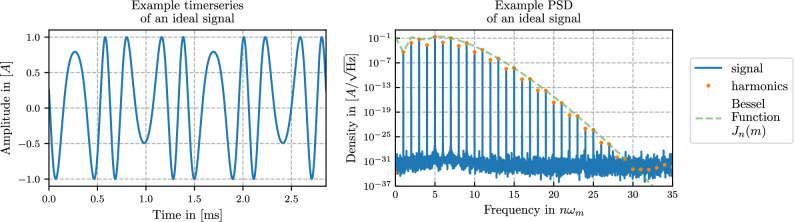
Time-series and power spectral density of an example ideal signal as defined in (1) with modulation index $m=7$. The frequency peaks are the individual parts of the Fourier series of the signal (3). The dotted line (here given by $J_n(7)$) is the enveloping function for the peaks. In this simulation, no additional noise besides the intrinsic digitization noise is present leading to a noise floor around $10^{-32}$. In a real experimental setup, this noise floor will be higher due to other additional noise sources which will ultimately limit how many harmonics can be resolved and used for the parameter estimation.

Throughout this article we refer to the individual parts of the Fourier series (2) and (3) as ’signal harmonics’ or harmonic frequencies. Figure 2 shows a time-series and the power spectral density of an example signal with these harmonics clearly visible as ’peaks’ in the spectrum.

For DFMI and similar techniques, the modulation index scales with the differential arm-length $\Delta L$. E.g. the modulation index is given by the product $m:= \Delta \omega \cdot \delta \tau$ of the modulation depth $\Delta \omega$ and propagation time-difference $\delta \tau$ (related to the arm-length difference via $\Delta L = c \cdot \delta \tau$, with *c* as speed of light)^10^. Hence, *m* encodes a macroscopic length in DFMI that is complementary to the microscopic length in $\varphi := \omega _0 \cdot \delta \tau$ (which is periodic and limited to $\varphi \in [0, 2\pi )$).

From (3) we see that even and odd harmonics behave like different quadratures of the phase $\varphi$. These ’quadratures’ contain Bessel functions which are not periodic and scale differently with changing path-length differences $\Delta L$. The Bessel functions are not invertible, meaning that there is no direct way to calculate the modulation index *m* (and the corresponding macroscopic path-length difference) for these measured harmonics. By use of a recurrence relation for the Bessel functions one can still calculate *m* from multiple harmonics to get the macroscopic distance and subsequently divide them out to reveal simpler trigonometric relations.

Most studied readout algorithms^10,12,13,15–17^ make use of the Fourier-series description ((2) or (3)), where they employ either a fast Fourier transform or a dedicated demodulation^22^ to determine the amplitude of a set of the signal harmonics and then apply further computations. These readout schemes realise the parameter estimation by approximating the signal^13,15,16^, the use of a numeric, non-linear fit algorithm^12^ and/or the use of a fixed modulation index^11,13,15,17^ (corresponding to specific absolute arm-length difference in DFMI).

## Analytic algorithm

The algorithm presented here operates on a set of signal harmonics which are demodulated individually. Then it uses a recurrence relation between the Bessel functions to provide an analytical estimate of the modulation index *m*. Knowing *m* we calculate the Bessel functions and extract them to isolate the interferometric quadratures. And with these quadratures, the phase estimate can be calculated. The algorithm can thereby calculate all parameters of interest without any initial assumption and in a non-iterative fashion for arbitrary input parameters. A sketch of the algorithms as flowchart can be seen in Fig. S.1 in the Supplementary Information with the letters following the names of the paragraphs in this section.

We assume the signal in (1) to be measured on a photo-diode, sampled and digitized with a sampling frequency of $f_S$, leading to a measured time series $s(t_1), \cdots , s(t_N)$ over a time period $T$ which is chosen to be an integer multiple of the modulation time $2\pi /\omega _m$.

### Initial coefficients from the measured signal

We assume that the modulation frequency $\omega _m$ is well known, if not it could be extracted from the FFT of the time-series, since the measured signal is a sum of ’harmonics’ frequencies $n \cdot \omega _m$ with $n \in {\mathbb {Z}}$.

#### Definition of $I_n$ and $Q_n$

The first step in our algorithm (similar to^10,12^) is to calculate the coefficients of the Fourier series of the signal. By I-Q-demodulation of the harmonics of the signal, we define the $I_n$ and $Q_n$ coefficients as:

4 $$\begin{aligned} I_n:= \frac{1}{T} \int _{0}^T dt \ s(t) \cdot \cos (n \omega _m t) \cdot W(t) \hspace{2cm} Q_n:= \frac{1}{T} \int _{0}^T dt \ s(t) \cdot \sin (n \omega _m t) \cdot W(t). \end{aligned}$$

The resulting coefficients of this demodulation are listed in Table 2. Besides the measured signal *s*(*t*) and the $\sin$ and $\cos$ factor of the demodulation, we apply a window function *W*(*t*) in (4) which acts as additional low-pass filter to suppress the leakage of the other harmonics of the discreetly sampled data.

**Table 2:** Table of the $I_n$ and $Q_n$ coefficients obtained from the I-Q-demodulation of the different DFM harmonics.

Low-pass filter of	$=:$	$n$ even	$n$ odd
$s(t) \cdot \cos (n \omega _m t)$	$I_n$	$\, \ \ A \cdot J_n(m) \cdot \cos \varphi \cdot \cos n\psi$	$-A \cdot J_n(m) \cdot \sin \varphi \cdot \sin n\psi$
$s(t) \cdot \sin (n \omega _m t)$	$Q_n$	$-A \cdot J_n(m) \cdot \cos \varphi \cdot \sin n\psi$	$-A \cdot J_n(m) \cdot \sin \varphi \cdot \cos n\psi$

#### Filtering of the demodulated signal (Regarding the window function *W*(*t*))

Choosing a measurement time *T* of multiples of $2\pi /\omega _m$ (corresponding to a rectangular window function $W_\text {rect}(t) = \text {rect}(t/T-1/2)$), already provides a major suppression for the other harmonics. The Fourier transform of such a window function

5 $$\begin{aligned} {\tilde{W}}_\text {rect}(\omega ) \propto \text {sinc}(\omega T / 2 \pi ) \end{aligned}$$

has zeros at $\omega = 2\pi /T \cdot k$ with *k* as integer and choosing $T = 2\pi /\omega _m$ ensures that all the other harmonics (with $k \ne 0$) are suppressed as shown in Supplementary Fig. S.2 in the Supplementary Information. For real signals, additional filtering may be necessary as the measurement time *T* or the modulation frequency $\omega _m$ might not exactly satisfy this condition, as described by Schwarze et al.^22^. In our reference implementation, we use a squared Hanning window

6 $$\begin{aligned} W(t) = \frac{4}{3T^2} \sin ^2(\pi t / T) \end{aligned}$$

(also shown in Fig. S.2) which provides a falloff to higher frequencies in the order of $\propto 1/f^5$. The integration operation in (4) acts as additional low-pass filter (which factors in as another 1/*f* in Fourier space).

### Calculating the modulation phase $\psi$

The calculation of the modulation phase $\psi$ is done identical to the algorithm by Heinzel et al.^12^. E.g. by calculating the arctan of the ratio $I_n/Q_n$ for the measured $n$’th harmonics and unwrapping the resulting values one obtains $\psi$. For completeness we write this as

7 $$\begin{aligned} {\left\{ \begin{array}{ll} \arctan (-Q_n/I_n) & \ \text {n even} \\ \arctan (I_n/Q_n) & \ \text {n odd.} \end{array}\right. } = {\left\{ \begin{array}{ll} n \psi \ \textrm{mod}\ \pi /2 & \ \text {for } n \psi \ge 0 \\ n \psi \ \textrm{mod}\ -\pi /2 & \ \text {for } n \psi < 0 \end{array}\right. } \end{aligned}$$

The resulting values are multiples of $\psi$ modulo $2\pi$. To get $\psi$, we add $\pi$ at jumps of the calculated list of $(n\cdot \psi \ \text {mod}\ \pi )$ and average the gradient.

### Definition of the $c_n$ coefficient

Next we eliminate the $\psi$ factor by calculating

8 $$\begin{aligned} c_n := {\left\{ \begin{array}{ll} +I_n \cdot \cos n\psi - Q_n \cdot \sin n\psi \quad \text {n even} \\ -I_n \cdot \sin n\psi -Q_n \cdot \cos n\psi \quad \text {n odd} \end{array}\right. } = {\left\{ \begin{array}{ll} A \cdot J_n(m) \cdot \cos \varphi \quad \text {n even} \\ A \cdot J_n(m) \cdot \sin \varphi \quad \text {n odd} \end{array}\right. } \end{aligned}$$

using the previously calculated estimate of $\psi$.

### Calculating the modulation index $m$

The crucial step in our algorithm is the estimation of the modulation index *m*. We make use of a recurrence relation (9) for Bessel-functions of the first kind that can be rearranged to yield *m*.

9 $$\begin{aligned} J_{n-1}(m) + J_{n+1}(m) = \frac{2n}{m} J_{n}(m) \quad \implies \quad m = \frac{ 2 n J_{n}(m) }{J_{n-1}(m) + J_{n+1}(m) } \quad \quad \text {for } m \in {\mathbb {Z}}. \end{aligned}$$

Since the harmonic amplitudes / the $c_n$ coefficients contain $\sin \varphi$ and $\cos \varphi$ factors, one can not directly plug them in (9). Hence we apply the recursive relation twice and solve for *m* to get the relation

10 $$\begin{aligned} m = \sqrt{ \frac{4 n (n-1) (n+1) J_n(m)}{2n J_{n}(m) + (n+1) J_{n-2}(m) + (n-1) J_{n+2}(m)} }. \end{aligned}$$

Plugging the $c_n$ coefficients obtained from (8) into this relation in place of the $J_n$, the $A$ and $\varphi$ dependant factors cancel out and only the $J_n(m)$ factors remain such that

11 $$\begin{aligned} m_n :=&\sqrt{ \frac{4 n (n-1) (n+1) c_n}{2n c_n + (n+1) c_{n-2} + (n-1) c_{n+2}} } = m \ \text {for} \ \forall \, n . \end{aligned}$$

Next, we average the calculated $m_n$ values from every set of 3 harmonics ($n-2, n, n+2$) to get the *m* estimate.

For noise dominated harmonics (leading to highly erroneous $c_n$), the values in the square-root of (11) is also highly erroneous and can even become negative. In this case we continue with the absolute value (to calculate any real value from the square root) and effectively discard the erroneous $m_n$ later by using a weight that takes the harmonics individual signal-to-noise ratio into account. Without any prior knowledge we would use e.g. the power of the harmonics as weight when averaging over the $m_n$ values. To achieve the highest possible precision it is however necessary to use a specific weighting function as specified in Sect. 3.7.

### Calculating the interferometric phase $\varphi$

Having calculated an estimate for $m$ before, we calculate the estimate of $\varphi$ straight forward by

3.5.1 Eliminating the *m* dependency from the $c_n$ coefficients via
  12 $$\begin{aligned} \frac{c_n}{J_n(m_\text {estimate})} = {\left\{ \begin{array}{ll} A \cdot \cos (\varphi ) \ \ \text {for n even}\\ A \cdot \sin (\varphi ) \ \ \text {for n odd} \end{array}\right. } \end{aligned}$$
3.5.2 Calculating a weighted average over the even and odd peaks (as explained in 3.7) leading to one value for each quadrature, $A \cdot \sin (\varphi )$ and $A \cdot \cos (\varphi )$ .
3.5.3 Similar to other interferometric phase readout procedures, we calculate the final estimate for $\varphi$ from the two quadratures via
  13 $$\begin{aligned} \varphi _\text {estimate} = \arctan \left( \frac{A \cdot \sin (\varphi )}{A \cdot \cos (\varphi )} \right) \end{aligned}$$
  It is common practice for interferometric measurements to use a atan2 routine (which compares the sign of the quadratures to extend the output range to $\varphi \in (-\pi , \pi ]$). The phase is then calculated from the quadratures via
  14 $$\begin{aligned} \varphi _\text {estimate} = \text {atan2}(\sin (\varphi ), \cos (\varphi )) \end{aligned}$$

Additionally, for continuous interferometric measurements, the phase is usually unwrapped using a ’phase-tracking’ algorithm. This further helps to resolve ambiguities at exactly multiples of $\pi /2$.

### Calculating the amplitude *A* and offset *B*

For a clean signal one can estimate the amplitude *A* and the offset *B* from the minimal and maximal values. A more elaborate method to estimate *A* is to extract an estimate for each harmonic and then to average using the optimal weights. Amplitude and offset are often required with less precision and are used to measure effects due to beam tilts that decrease the optical contrast and the constant optical power impinging on each photodiode or a segment of the same.

### Averaging over individual peak values

In the algorithm, (almost) every harmonic yields an estimate for *m*, $\varphi$ and $n\psi$. To mitigate noise at specific frequencies and to take the individual SNR of the harmonics into account, we found that a crucial part of achieving optimal performance is to perform a weighted average over these values.

A simple weight is to directly use the signal power of the individual harmonics $|{\tilde{s}}(n\omega _m)|^2$, which is proportional to $\propto N \cdot c_n^2$. For the calculation of the $m_n$ which involves three harmonics (with $n-2$, *n* and $n+2$), we use the product of the signal power of these three harmonics as weight for the n’th harmonic. To achieve optimal performance, we use however a different weight given by

15 $$\begin{aligned} w_n = \frac{2 |J_n(m)|}{m} \left( 1 + \frac{m^2 \left( 1 + \frac{1}{2n} \right) }{(n^2 -1)} \right) ^{-1} \cdot |{\tilde{s}}(n\omega _m)|^2 \end{aligned}$$

which is the result of the error-propagation of equation (10) as derived in in Sect. 4.1 and an additional $|{\tilde{s}}(n\omega _m)|$ factor. This additional $|{\tilde{s}}(n\omega _m)|^2$ factor accounts for the individual signal-to-noise ratio of the harmonics. At certain phase values (e.g. $\varphi \approx 0$ or multiples of $\pi /2$), all even (or odd) harmonics contain very little signal energy. In such a case the remaining other odd (or even) harmonics contain most of the signal energy and need to be weighted higher.

A problem of the ideal weights (15) is that they themselves depend on the modulation index *m*. If no prior information is available; we run the algorithm twice, with the first iteration using only the harmonics signal power as weight; and further iterations use the resulting *m* value from previous iterations to calculate the weighting function, using the same data for every iteration. In an experimental setup with continuous measurements, we use the values from the previous run to calculate the weights so that the averaging of the $m_n$ coefficients only happens once.

## Algorithm performance and error analysis

The use case of our algorithm in DFMI has (in general) 5 free parameters ($B, A, m, \varphi , \psi$) with $\omega _m$ being fixed. Our algorithm should be able to deal with arbitrary real values for any of these parameters. In an experimental setup, the parameter space is however restricted to certain ranges. E.g. the sampling and modulation frequency limit the number of resolvable harmonics and any present noise will degrade the readout results.

A more extensive study on the maximally achievable readout performance for various additive white noise contributions can be found in^18^. The Cramer–Rao lower bound (CRLB) derived there will serve as optimal goal for our algorithms performance analysis. To test the readout algorithm, we simulate a signal with the same parameters as Gerberding et al.^10^ (including the additive white noise in the order of $\sigma = 2 \cdot 10^{-7}$) and let the algorithm calculate the parameters. From the equations derived in Eckhardt & Gerberding^18^ (as shown in (18) as amplitude spectral density) we can calculate what the minimal achievable noise floor for the individual signal parameter estimates is (the CRLB).

16 $$\begin{aligned} \text {CRLB}_m (f)&= \frac{\sqrt{8} \sigma }{f_S N A \sqrt{ 3 + 4 J_0(m) \cdot \cos (\varphi ) + J_2(2m) \cdot \cos (2\varphi ) }} \end{aligned}$$

17 $$\begin{aligned} \text {CRLB}_\psi (f)&= \frac{4 \sigma }{f_S N A \sqrt{m^2 - m \cdot J_1(2m) \cdot \cos (2 \varphi ) }} \end{aligned}$$

18 $$\begin{aligned} \text {CRLB}_\varphi (f)&= \frac{\sqrt{8} \sigma }{f_S N A \sqrt{ 1 - J_0(2m) \cdot \cos (2 \varphi )}} \end{aligned}$$

**Fig. 3:**
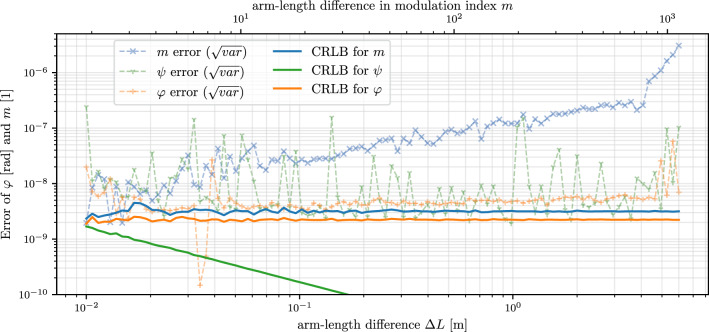
Absolute error of the new readout algorithm for varying arm-length difference $\Delta L$ for a simulated DFMI setup. The x-axis is shown once in absolute arm length difference $\Delta L$ (lower axis) and once in modulation index $m = \Delta \omega /c_0 \cdot \Delta L$ as it appears as signal parameter. The other signal parameters are the phase $\varphi = 2\pi \Delta L/\lambda$ and $B=1$, $A=1$, $\psi ={0.1}\text {rad}$, $\Delta f = {9}\text {GHz}$, $\lambda = {1064}\text {nm}$, $f_S = {2}\text {MHz}$, $f_m = {1}\text {kHz}$, $f_R=1/T={100}\text {Hz}$ and some added Gaussian noise in the order of $\sigma \approx 2 \cdot 10^{-7} \text {V}/\sqrt{\text {Hz}}$. For each datapoint in the plot we ran the algorithm 100 times with the same signal but with varying noise and calculated the variance. The initial distance value (with $m \approx 2$) corresponds to a signal where only a 3 harmonics are visible in the frequency spectrum while the last value ($m \approx 1131$) corresponds to the measurement band being filled with harmonics up to the Nyquist frequency (e.g. $1131 \cdot f_m \gtrapprox f_s/2$). Since no anti-aliasing filter was applied the noise increase above ($m \approx 700$) is dominated by aliased harmonics.

Figure 3 shows the result of this simulation for varying distance $\Delta L$ between 1cm and 6m, leading to different modulation indices *m* and phases $\varphi$ respectively. For almost the entire range, the phase $\varphi$ reaches close (smaller than $\times 2$) to the CRLB. At the ’edges’ other effects start to influence the precision. For too small *m*, not enough harmonics are visible above the noise floor and the algorithm cannot properly run since it needs at least 6 harmonics (3 even and 3 odd) for its calculations. If there are just enough harmonics visible to run the algorithm, a single harmonic with a bad signal-to-noise ratio degrade the performance as they are not averaged out by other higher order harmonics. For too large *m*, the bandwidth of the measurement (set by the sampling frequency) is filled with harmonics and higher-order harmonics start to spoil via the alias-effect the lower-order harmonics. Increasing the sampling frequency in simulations resolves this issue; in an experimental setup an anti-alias filter (as they are commonly applied before ADCs) similarly mitigates this effect.

### Error approximation for *m*

**Fig. 4:**
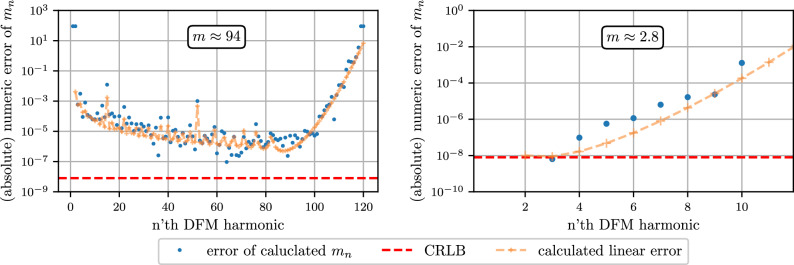
Absolute error of calculated $m_n$ values for a simulated signal with the same parameters as in Fig. 3, once with $m \approx 94$ (distance $\approx 0.5$ meter) on the left side and once with $m \approx 2.8$ on the right side. The upper error prediction (yellow dashed lines) correlates moderately well with the measured error (blue dots). (The error prediction is given by (21) with $\sigma = 2 \cdot 10^{-7}$ as before and an additional scaling factor of $f_m/f_S$.

When calculating the *m* parameter as outlined in Sect. 3.4 but without using the weighted averaging, the resulting error of the $m_n$ estimates can be many orders of magnitude above the CRLB as seen in Fig. 4.

Due to the linearity of the equations, any white noise in the signal will lead to the same white noise level for the $I_n$, $Q_n$ and $c_n$ coefficients. For the $m_n$ coefficients calculated from equation (10), the linear error propagation, when adding the errors ($\delta c _{n-2}, \delta c _{n},\delta c _{n+2}$), yields:

19 $$\begin{aligned}&m_n(J_{n-2}(m) + \delta c_{n-2}, J_{n}(m) + \delta c_n, J_{n+2}(m) + \delta c_{n+2}) \approx m + \sum _{k \in \{n-2, n, n+2\}} \frac{\partial m_n}{\partial J_{k}(m)} \cdot \delta c_{k} + {\mathcal {O}}(\delta c^2) \end{aligned}$$

20 $$\begin{aligned}&= m + \frac{m}{8 J_n(m)} \bigg [ 4 \delta c_n - \frac{m^2}{n(n^2-1)} \big ( (n-1)\delta c_{n+2} + 2n\delta c_{n} + (n+1)\delta c_{n-2}\big ) \bigg ] \end{aligned}$$

21 $$\begin{aligned}&\lesssim m + \frac{m}{2 |J_n(m)|} \bigg [1 + \frac{m^2 \left( 1 + \frac{1}{2n} \right) }{(n^2-1)} \bigg ] \cdot \delta c \end{aligned}$$

When approximating the upper error in (21) we simplify by assuming that $\delta c_k \in [-\delta c, \delta c]$, which allows us to calculate a simple upper bound for the error. Figure 4 also shows the upper error as yellow dashed line which shows good agreement with the measured errors of the given sample. We find that this linear error estimation already yields a good estimate for the precision of the $m_n$ values. Which is why we use the inverse of this error estimator as weight for the individual harmonics as mentioned in (3.7).

In (21) we see that the error of our *m* estimate scales with *m* and $m^3$ (ignoring the $|J_n(m)|$ scaling for now) suggesting that small values for *m* result in smaller errors. The plots in Fig. 4 shows the error of the $m_n$ coefficients once for a large *m* value (left plot) and once for a small value (right plot). In case of a small *m*, the signal energy is distributed between fewer harmonics compared and the individual harmonics can have a smaller relative error close to the CRLB. The average is however roughly the same for both cases, indicating that there is no general favorable parameter region for *m*.

### Error approximation for $\varphi$

**Fig. 5:**
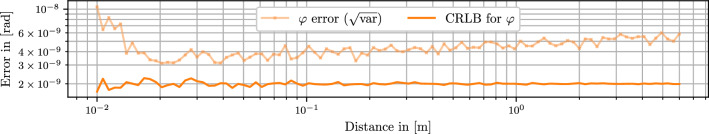
Absolute error of calculated $\varphi$ estimates for varying distances with the same parameters as in Fig. 3 but with the exact *m* parameter given before calculating $\varphi$.

Figure 5 is the result of running the readout algorithm with the same parameters as in Fig. 3 but giving the algorithm the exact *m* and $\psi$ values before calculating the $\varphi$ parameter. The resulting error is almost the same as in Fig. 3 where the exact parameters were not given before. The increasing error at the edges of the plot can be similarly explained by insufficient harmonics for proper averaging for small *m* and noise due to aliasing of higher order harmonics for large *m*. In between, the phase error remains around $2\times$ the CRLB.

Since the CRLB is the best achievable limit for the given (additive) white noise; we believe the remaining error comes from non-ideal filtering of the higher order harmonics (with power above the white noise) which can leak and add further “noise” to the other $I_n, Q_n$ and $c_n$ coefficients. This noise depends on the implementation of the filtering and a stronger filter might improve the results.

## Signal dynamics

In this section we consider the effects of a linear phase term $\delta \omega \cdot t$ and its effect on the readout algorithm. Such terms can appear i.e. when the mean frequency $\omega _0$ of a DFMI laser is not constant, but drifts over time, or when the target motion is so dynamic that the signal is Doppler shifted by the frequency $\delta \omega$. This breaks with the static signal assumption used in some of the previously studied readout algorithms.

We write the dynamic signal as

22 $$\begin{aligned} s_{\text {moving}}(t)&:= B + A \cos \left( m \cdot \sin \left( \omega _m t + \psi \right) + \varphi + \delta \omega \, t \right) \end{aligned}$$

23 $$\approx B + A \sum _{n \in {\mathbb {Z}}} J_n(m) \cos \left( (n\omega _m + \delta \omega ) t + n \psi + \varphi \right).$$

In a DFMI setup, a target moving with speed *v* would cause a Doppler shift of the signal of $\delta \omega = 2\pi /\lambda _0 \cdot v$. Similarly, the modulation index *m* also changes to $m \mapsto m + \Delta \omega v / c \cdot t$ with *v* being the target speed and *c* as speed of light. For small enough speeds *v* this additional time dependant term is negligible. For large signal dynamics it can become relevant for the estimation of *m* and influence the parameter estimation of the other coefficients. When simulating dynamic DFM signals for this publication, we always used the full $m + \Delta \omega v / c \cdot t$ expression for the signal.

**Fig. 6:**
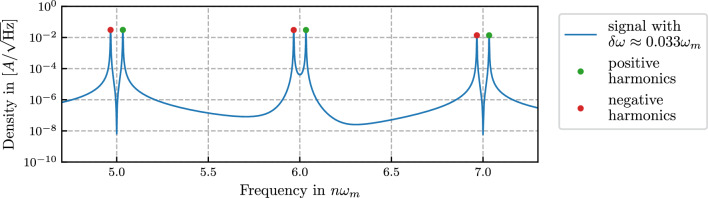
Signal with ’small’ ($\delta \omega < \omega _m/2$) frequency shift. The positive and negative frequency parts of the signal harmonics become visible and do no longer overlap.

For $\delta \omega = 0$ the parts in the sum of (23) with positive and negative indices have the same frequencies, leading to overlapping signals for every $n\omega _m$ harmonic. For $\delta \omega \ne 0$ this is no longer true as the spectrum appears shifted in the direction of $\delta \omega$. For small enough $\delta \omega$, the harmonics in the PSD appear to split into one peak belonging to the positive indices/frequencies ($n\omega _m + \delta \omega$) and one to the negative indices/frequencies ($-n\omega _m + \delta \omega$) (which are folded onto the positive region when calculating the PSD) as shown in Fig. 6

For the algorithm to work in the presence of a large Doppler shift, an estimator for $\delta \omega$ is needed to (a) demodulate the DFM harmonics at their correct frequencies ($n\omega _m + \delta \omega$) and (b) to account for the additional phase shift $\delta \omega T$ that is added to $\varphi$ over the course of the measurement period *T*. So instead of a single demodulation for each harmonic we implement two that track the splitting tones. The calculation of the parameters and coefficients used in the algorithm change then slightly compared to the previously shown calculation and is shown in Sect. 5.1. In the following we only consider the case where Doppler-shifts are smaller than half of the modulation frequency $\delta \omega \le \omega _m/2$. Our solution to deal with frequency shifts $\delta \omega \le \omega _m/2$ in continuous measurements is to initially run the algorithm, without any corrections, and then calculate the gradient of the resulting phase values and use it to generate a linear prediction of the frequency shift $\delta \omega$.

### Dynamic readout algorithm (for large $\delta \omega$)

For dynamic signals with $\delta \omega \ne 0$, the algorithm can be modified to account for the individual tones ($n\omega _m + \delta \omega$ and $n\omega _m - \delta \omega$) which were treated as single harmonic before. (Supplementary Fig. S.4 shows a sketch of the demodulation scheme that calculates the additional coefficients for a single (*n*’th) harmonic). Demodulating at exactly ($n\omega _m + \delta \omega$) and ($n\omega _m - \delta \omega$) yields slightly different *I* and *Q* coefficients ($\mapsto I_{n,\pm } \text { and } Q_{n,\pm }$) as defined in (24) and (written in Table S.1 and S.2 in the Supplementary Information).

24 $$\begin{aligned} I_{n,\pm } := \frac{1}{T} \int _{0}^T dt \ s(t) \cdot \cos ((n \omega _m \pm \delta \omega )t) \cdot W(t) \nonumber \\ Q_{n,\pm } := \frac{1}{T} \int _{0}^T dt \ s(t) \cdot \sin ((n \omega _m \pm \delta \omega )t) \cdot W(t) \end{aligned}$$

In the limit of $\delta \omega \mapsto 0$, the $Q_{n,\pm }$ and $I_{n,\pm }$ coefficients converge to the previously introduced $I_n$ and $Q_n$ coefficients from Table 2.

Using these coefficients and the additional derived coefficients displayed in Table S.2 (in the Supplementary Information), the algorithms calculation of the individual parameters changes slightly:

5.1.1 for $\psi$, instead of calculating the $Q/I$, we calculate $(Q_{n,+} + Q_{n,-}) / (I_{n,+} + I_{n,-})$ and continue with the result as before
5.1.2 for $m$, we calculate the $\psi$-free $c_{n,+}$ and $c_{n,-}$ coefficients as
  25 $$\begin{aligned} c_{n,\pm }:= {\left\{ \begin{array}{ll}-(Q_{n,+} \pm Q_{n,-}) \cos (n\psi ) - (I_{n,+} \pm I_{n,-}) \sin (n\psi )\\ \quad \text {for } n \text { even}\\ \\ -(Q_{n,+} \pm Q_{n,-}) \sin (n\psi ) + (I_{n,+} \pm I_{n,-}) \cos (n\psi )\\ \quad \text {for } n \text { odd} \end{array}\right. } \end{aligned}$$
  and plug the $c_{n,+}$ coefficients into equation (11) to continue as before
5.1.3 To obtain the $\varphi$ estimate, we first eliminate the remaining *m* (and *n*) dependency in the $c_{n,\pm }$ coefficients by calculating
  26 $$\begin{aligned} d_{\pm ,n}:= \frac{2 c_{n,\pm }}{A J_n(m)}, \end{aligned}$$
  which yields for even and odd *n*:
  27 $$\begin{aligned} d_{+,n_\text {even}}&= \cos \varphi + \cos (\delta \omega T + \varphi ) \text {sinc}(\delta \omega T) \nonumber \\ d_{-,n_\text {even}}&= \sin \varphi - \sin (\delta \omega T + \varphi ) \text {sinc}(\delta \omega T) \nonumber \\ d_{+,n_\text {odd}}&= \sin \varphi + \sin (\delta \omega T + \varphi ) \text {sinc}(\delta \omega T) \nonumber \\ d_{-,n_\text {odd}}&= \cos \varphi - \cos (\delta \omega T + \varphi ) \text {sinc}(\delta \omega T) \end{aligned}$$
  Next, we average these coefficients over all even and odd harmonics using our custom weights, leading to exactly 4 averaged coefficients $d_{+,\text {even}}, d_{+,\text {odd}}, d_{-,\text {even}}, d_{-,\text {odd}}$. From these 4 coefficients we calculate the phase via:
  28 $$\begin{aligned} \varphi := \arctan \left( \frac{d_{+,\text {odd}} + d_{-,\text {even}}}{d_{+,\text {even}} + d_{-,\text {odd}}} \right) \end{aligned}$$

### Precision in case of a (linear) frequency shift $\delta \omega$

**Fig. 7:**
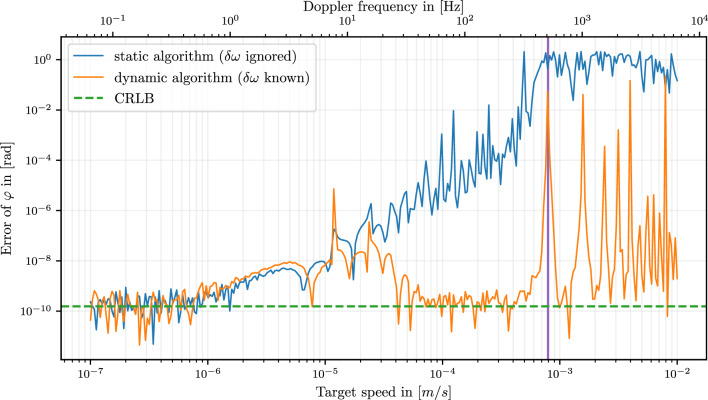
Algorithm performance for modeled DFM signal ($B=0,A=1,\psi =\pi /4, f_s = 524 \ \text {kHz}, f_m = 1 \ \text {kHz}$, $f_R = 16 \ \text {Hz}$, $\lambda = {1550}\text {nm}$ and initial distance $L_0 = {20}\text {cm}$) with additive white noise ($\sigma = 2 \cdot 10^{-7}$) and varying relative frequency shifts $\delta \omega = 2\pi v/\lambda$. For each point in the plot, we simulated a target moving with constant speed 100 times (but always starting at the same position $L_0$) and averaged the result. The blue line shows the readout algorithm result in case this Doppler shift is ignored and the algorithm runs as described in Sect. 3. The orange line shows the readout algorithm result in case the Doppler shift is known before and algorithm can account for it as explained in Sect. 5.1. The dashed green line is the CRLB showing the highest reachable precision. The vertical purple line marks a Doppler shift of $f_m / 2$ (here at $500 \ \text {Hz}$). At this point two neighboring harmonics (e.g. $n\omega _m + \delta \omega$ and $(n+1)\omega _m - \delta \omega$) overlap and the demodulation of a single isolated harmonic is not possible, leading to the algorithm failing at exactly multiples of $f_m/2$.

Figure 7 shows the simulated performance of the readout algorithm for different relative frequency shifts $\delta \omega$ between two consecutive measurements for DFM signals as given by

29 $$\begin{aligned} s_{\text {DFM, moving}}(t)&= B + A \cos \left( m(t) \cdot \sin \left( \omega _m t + \psi \right) + \varphi + \delta \omega \, t \right) \end{aligned}$$

30 $$= B + A\sum\limits_{{n \in \mathbb{Z}}} {J_{n} } (m + \delta m \cdot t) \cdot \cos \left( {(n\omega _{m} + \delta \omega )t + n\psi + \varphi } \right){\text{ }}$$

with $$m(t):= m + \Delta \omega \frac{v}{c} \cdot t =: m + \delta m \cdot t$$.

For Doppler shifts below $\approx 0.6\text { Hz}$ the target is moving so slow that the algorithm consistently reaches the CRLB (green dashed line). For larger Doppler shifts $> 50\text { Hz}$ the extended algorithm (Sect. 5.1) is still able to achieve close to optimal precision (orange line) while not accounting for the Doppler shift will yield gradually worse results (blue line).

For the ’bump’ between 0.6 and 50 Hz, where the ’static’ and ’dynamic’ algorithm yield the same result but do not reach the CRLB, we found three contributing factors of similar size leading to the higher noise.

Firstly, insufficient filtering during demodulation. Since the harmonics are shifted by $\delta \omega$, they no longer ’sit’ exactly at the zeros of our filter (as described in Sect. 3.1.2) and start to leak into the calculation of the $I_n$ and $Q_n$ coefficients of their neighboring harmonics.

Secondly; the filtering of the splitted tones for the dynamic algorithm. During demodulation of the splitted tones, small enough Doppler shifts are not filtered out sufficiently (like the higher order harmonics) and perturb the calculated $I_{n,\pm }$ and $Q_{n,\pm }$ coefficients. This is why after the initial decrease in precision (after $\approx 1 \text { Hz}$ in Fig. 7), the precision increases again for the dynamic algorithm as the splitted tones are separated more clearly and filtered out during demodulation of the other tone respectively.

The third effect comes from the DFM signal harmonics containing an additional time dependency in the *m*(*t*) parameter. We were yet unable to calculate a closed expression for the $I_n$ and $Q_n$ coefficients when writing the signal with the exact time dependence including $m(t) = m_0 + \Delta \omega v t / c$. Even with perfectly filtered harmonics, the demodulated harmonic itself has an additional phase error proportional to $\delta m$ that we disregarded in our algorithm so far.

### Example readout for an oscillating mass

**Fig. 8:**
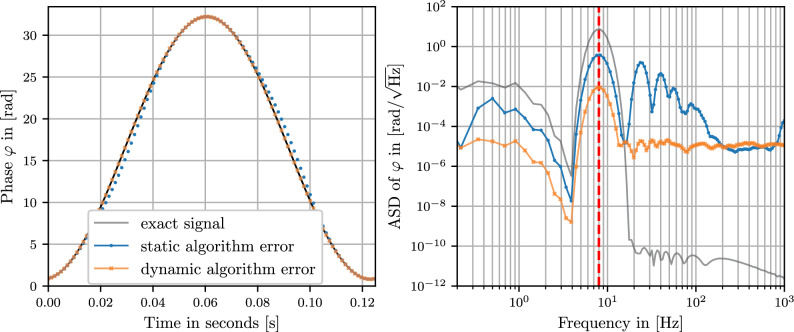
Algorithm results for a simulated moving target, oscillating with a frequency of 8 Hz and an amplitude of 2.5 wavelengths (a total path of 5 wavelengths). The DFM signal ($f_m \approx 1 \text { kHz}$) was sampled with $f_S = 2 \text { MHz}$ and a readout frequency of $f_R \approx 8 \text { kHz}$ and otherwise the same parameters and additive noise ($\sigma = 2 \cdot 10^{-7}$) as in Fig. 7 . The black line marks the exact phase of the target. The blue line is the algorithm output as written in Sect. 3 (without the dynamic extension) and the orange line is the result when using the dynamic extension detailed in Sect. 5.1. The left plot shows one period of the resulting time-series and the right plot shows the amplitude spectral density calculated with an LPSD algorithm^23^. The dashed red line marks the frequency of the movement of the oscillating target.

As more realistic example of a measurement, Figure 8 shows the results of the readout algorithm for an oscillating target. Its speed varies over time and the movement contains not only linear but also higher order terms in *t*. From the time-series (Fig. 8, left plot) we can clearly see that at the points of maximum slope (when the linear speed term is largest and all others small), the ’static’ algorithm version has the largest error, while at the turning points, the deviation from the exact phase is similarly for both ’static’ and ’dynamic extension’ of the algorithm.

It should be noted that the difference in the ASD plot (Fig. 8, right plot) between the static and the dynamic algorithm version is only around a factor of 40 while Fig. 7 shows a difference of around 5 orders of magnitude for the maximum speed of $\approx {125}{\upmu \text {m} / \text {s}}$ (a Doppler shift of $\delta \omega = 2\pi \cdot 125 \text { Hz}$) of the simulated target. The difference between Figs. 7 and 8 is that Fig. 7 is the result of a purely linear movement. This is where the difference between static and dynamic algorithm is greatest. The simulated signal for Fig. 8 has periods of very slow movement, here the static algorithm is just as precise as the dynamic one. Additionally, the movement of Fig. 8 is non-linear and relatively fast and strong compared to the modulation frequency, which causes errors in both the ’static’ and ’dynamic’ algorithm version that are not accounted for, leading again to a similar error in both versions. More optimal implementations of the dynamic algorithm that take into account not only linear speed might mitigate these errors further.

## Conclusion and outlook

For the interferometric phase $\varphi$ our new algorithm achieves close to optimal performance, within a factor of 2 or better of the CRLB. For the modulation index *m*, our analysis of the linear error propagation shows that it can have an error up to $\approx 10^2 \times \text {CRLB}$ for large *m* values. The lowest errors for estimating *m* of $\lessapprox 10 \times \text {CRLB}$ are achieved for $m<20$.

Besides the basic algorithm in Sect. 3, we provide an extended version detailed in Sect. 5.1 to deal with very dynamic signals (with large Doppler shifts). By using adaptive/dynamic filters it might even be possible to use the algorithm for even larger dynamics than shown in this paper. The precision achieved with our reference implementation does not use any initial values. Especially for larger Doppler shifts ($\delta \omega$) it is possible to use the algorithm e.g. with a close initial estimate for $\delta \omega$, run the algorithm and then calculate a more accurate and potentially larger $\delta \omega$ from the result. As long as the frequency difference between this initial estimate and the true value is small enough, even much larger absolute Doppler shifts can be accounted for. For future improvements we aim to add a scheme to improve the algorithms precision over time by, for example, deploying a Kalman filter for the Doppler-shift estimation.

Real DFMI setups contain additional noise sources like 1/*f* laser frequency noise. The influence of the most relevant of such noise sources have been analysed shortly in the Supplementary Material. In the case of non-white additive noise or the presence of parasitic tones one may adjust the weights of the algorithm to optimise the available SNR. Stray light and ghost beams lead to additional unwanted DFM-like signals overlapping with the ideal signal and potentially cause additional errors. An actual interferometer application also has additional requirements which will need to be met. This includes the ability to provide real-time estimates to run stabilisation control loops. E.g. to lock the average laser frequency to a reference interferometer^1^ and being able to cope with the presence of parasitic ghost beams^24^. The latter will require an extension of the algorithm in the presence of more than one beat note. This can be supported by utilizing additional information provided by the readout of two complementary photodiodes at the two outputs of an interferometer, so called balanced detection. In the case of balanced detection or when using quadrant photodiodes to also implement a beam-tilt readout via differential wavefront sensing, the readout of multiple channels can be further optimised, because they will share many of the parameters to be estimated, similar to the optimisation of a phase-locked-loop for heterodyne interferometry by Heinzel et al.^25^. The core scheme of the algorithm, the estimation of the modulation depth, will also be possible when multiple overlapping signals are present, but it will require either more a-priori parameter knowledge or additional estimation steps.

Lastly, our current implementation is purely sequential and does not use any parallelization of the individual algorithm steps. Unlike an iterative fitting of the signal, the algorithm allows for parallelization e.g. by performing the calculation steps for the used harmonics in parallel. Further speed can be gained by implementing the computational expensive but repetitive calculations of the *I* and *Q* coefficients into an FPGA, as they involve a large number of additions and multiplications with constant factors. Using much higher modulation and demodulation frequencies, or achieving higher rates of phase estimation and readout frequency $f_R$ to deal with more dynamic signals also requires a less computationally expensive and therefore faster readout algorithm, as presented here.

The algorithm we present will be crucial in the further deployment and development of compact interferometric sensors using DFMI. It relies only on simple analytical calculations, has a deterministic processing time and operates close to optimal noise performance. The analytic nature of the algorithm also make it easily compatible with other readout schemes to provide either initial parameter estimates or to get a low noise parameter estimation, depending on the specific use case.

## Supplementary Information

Supplementary Information.

## Data Availability

The datasets generated and analyzed for this publication are available from the authors on reasonable request at tobias.eckhardt@physik.uni-hamburg.de.
