# Supplementary material for: An analytic, efficient and optimal readout algorithm for compact interferometers based on deep frequency modulation
Source: Sci Rep. 2024 Sep 23;14:21988. doi: 10.1038/s41598-024-70392-9 (PMC11420234; doi:10.1038/s41598-024-70392-9)
Supplement: Supplementary file 1 — Supplementary Information. [file 41598_2024_70392_MOESM1_ESM.pdf]

S Supplementary material

S.1 Flowchart of the analytic algorithm

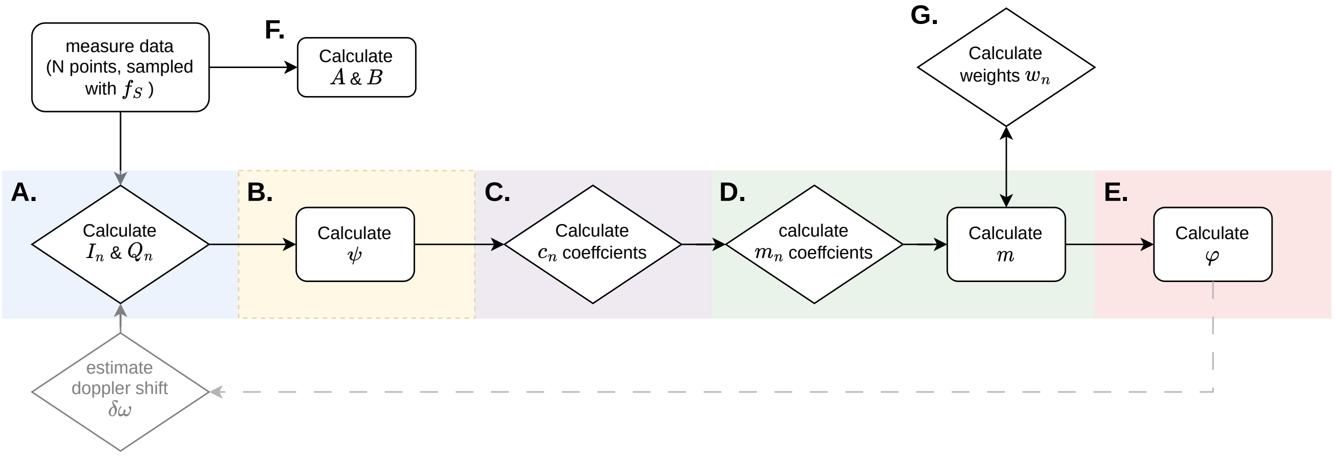

Figure S.1. Flowchart of the analytic algorithms. The grey dashed lines correspond to additional calculation steps explained in Appendix 5.1 for the case of a dynamic signal.

S.2 Window function Plot

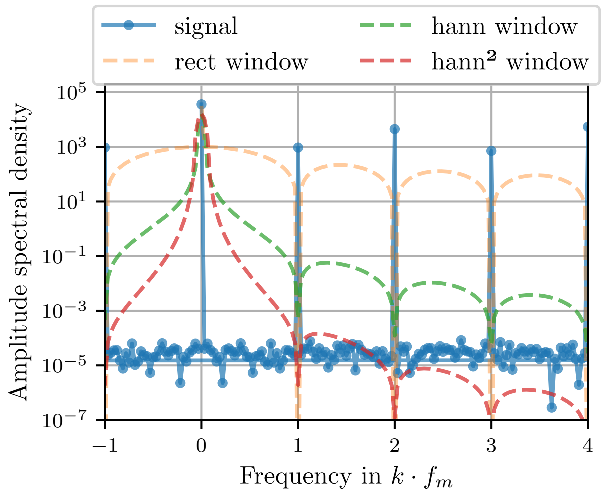

Figure S.2. Fourier transform of the signal and different window functions for one modulation period ($T = 2\pi/\omega_m$). In the I-Q-Demodulation scheme, the demodulated expression must be low-pass filtered (in this case by applying a window function and integrating the time-series) to suppress the contribution of the $k \neq 0$ harmonics. The window functions shown here all have zeros at multiples of the modulation frequency ω_m.

S.3 Algorithm relations to Deep-Phase Modulation (DPM) Inferometry

The predecessor of DFMI, Deep-Phase modulation interferometry (**DPMI**) introduced by Heinzel[12] has an almost identical signal to DFMI. In a DPMI setup as shown in Figure S.3, the phase modulation index m does not scale with the interferometers differential arm-length ΔL. Here a sinusodial modulation is introduced to the signal with the intend to create higher order harmonics, which also carry the microscopic distance / the phase information of the arm-length difference. When the differential arm-length changes, the modulation index remains constant. The analytic readout algorithm we present here works however the same on both DFMI and DPMI signals. Only the physical interpretation on the calculated coefficients differs.

S.4 Dynamic Readout Extension

Figure S.4 shows a sketch of the demodulation scheme for the splitted tones / harmonics of a dynamic signal as introduced in section 5. Here the frequency shift $\delta\omega$ must be known in the beginning of the algorithm to demodulate the harmonics correctly

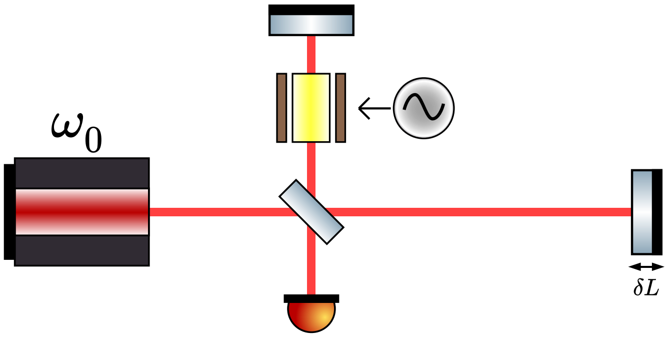

Figure S.3. Sketch of a DPMI setup. Here, only the relative changes of the arm-length difference / the test-mass position can be measured with high precision.

at their shifted frequencies.

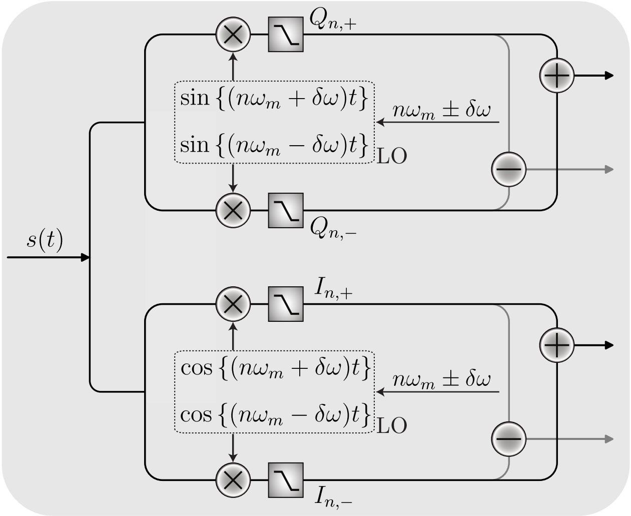

Figure S.4. Sketch of the demodulation scheme of the n'th harmonic. In the algorithm, this part is present multiple times up to the number of harmonics visible above the noise floor.

S.5 A remark about estimating $\delta\omega$

A general solution for getting a value of $\delta\omega$ would be to run a phase-locked loop (PLL) to track the frequencies of the DFM/DPM harmonics which are $n\omega_m \pm \delta\omega$. Due to the unique structure of the signal, most common PLLs which track a single frequency will however not work here.

PLLs construct an error signal (as measure of distance to the correct phase or frequency value) by comparing the measured signal to a locally generated one. In most cases the amplitude (or rather signal energy) factors into this errorsignal (e.g. it does so for the I and Q values from I-Q-demodulation and also when calculating any cross-correlation between signals). For pure sines or cosines, the amplitude and phase of their Fourier transform are independent from one another in Fourier domain. (A change in phase alone does not significantly affect the amplitude when calculating the PSD). For our signal this is not true as the amplitude of a single harmonic also varies with the φ and ψ parameters. E.g. while the total energy of the signal does not change when changing theses parameters, the distribution of the total energy between the different harmonics does change. For a PLL to work this means that it needs to track at least multiple harmonics of the signal.

We found that the highest precision for tracking a frequency shift $\delta\omega$ can be obtained when comparing the measured signal to a similar locally generated one (which includes all of the signal harmonics):

$$s_{\text{NCO}}(t) = \cos\left(m \cdot \sin\left(\omega_m t\right) + \omega_{\text{NCO}} t\right) \tag{1}$$

Calculating i.e. the cross-correlation between (1) and the measured signal yields a sufficiently good error signal. The downside is however that one would already need sufficiently good estimates for all the other parameters to construct such a local signal.

Such a PLL tracking would therefore have to be implemented in a control loop with the readout algorithm, as the output of one acts as input for the other component and is more complex than the solution we present in the following. It might, however, be relevant and useful for applications with more dynamic signals.

S.5.1 Additional coefficients used in the dynamic readout

The modified I_n and Q_n coefficients for the dynamic readout can be seen in Table S.1. From these, additional intermediate coefficients are calculated an seen in Table S.2 which are used to calculate the signal parameters as outlined in section 5.1.

low-pass filter of	=:	
$s(t)\cdot\sin((n\omega_m+\delta\omega)t)$	$Q_{n,+}$	$\frac{A}{2}\cdot J_n(m)\cdot\big[-\big(\sin\varphi-(-1)^k\sin(\delta\omega T+\varphi)\mathrm{sinc}(\delta\omega T)\big)\cos(k\psi)$ $-\big(\cos\varphi+(-1)^k\cos(\delta\omega T+\varphi)\mathrm{sinc}(\delta\omega T)\big)\sin(k\psi)\big]$
$s(t)\cdot\sin((n\omega_m-\delta\omega)t)$	$Q_{n,-}$	$(-1)^k\cdot\frac{A}{2}\cdot J_n(m)\cdot\big[+\big(\sin\varphi-(-1)^k\sin(\delta\omega T+\varphi)\mathrm{sinc}(\delta\omega T)\big)\cos(k\psi)$ $-\big(\cos\varphi+(-1)^k\cos(\delta\omega T+\varphi)\mathrm{sinc}(\delta\omega T)\big)\sin(k\psi)\big]$
$s(t)\cdot\cos((n\omega_m+\delta\omega)t)$	$I_{n,+}$	$\frac{A}{2}\cdot J_n(m)\cdot\big[-\big(\sin\varphi-(-1)^k\sin(\delta\omega T+\varphi)\mathrm{sinc}(\delta\omega T)\big)\sin(k\psi)$ $+\big(\cos\varphi+(-1)^k\cos(\delta\omega T+\varphi)\mathrm{sinc}(\delta\omega T)\big)\cos(k\psi)\big]$
$s(t)\cdot\cos((n\omega_m-\delta\omega)t)$	$I_{n,-}$	$(-1)^k\cdot\frac{A}{2}\cdot J_n(m)\cdot\big[+\big(\sin\varphi-(-1)^k\sin(\delta\omega T+\varphi)\mathrm{sinc}(\delta\omega T)\big)\sin(k\psi)$ $+\big(\cos\varphi+(-1)^k\cos(\delta\omega T+\varphi)\mathrm{sinc}(\delta\omega T)\big)\cos(k\psi)\big]$

Table S.1. Table of the I_n and Q_n coefficients obtained from the I-Q-demodulation of the different DFM harmonics for non-negligible frequency shifts $\delta\omega$.

	n even	n odd
$(Q_{n,+}+Q_{n,-})$	$-AJ_k(m)\Big(\cos\varphi+\cos(\delta\omega T+\varphi)\mathrm{sinc}(\delta\omega T)\Big)\sin(k\psi)$	$-AJ_k(m)\Big(\sin\varphi+\sin(\delta\omega T+\varphi)\mathrm{sinc}(\delta\omega T)\Big)\cos(k\psi)$
$(I_{n,+}+I_{n,-})$	$AJ_k(m)\Big(\cos\varphi+\cos(\delta\omega T+\varphi)\mathrm{sinc}(\delta\omega T)\Big)\cos(k\psi)$	$-AJ_k(m)\Big(\sin\varphi+\sin(\delta\omega T+\varphi)\mathrm{sinc}(\delta\omega T)\Big)\sin(k\psi)$
$(Q_{n,+}-Q_{n,-})$	$-AJ_k(m)\Big(\sin\varphi-\sin(\delta\omega T+\varphi)\mathrm{sinc}(\delta\omega T)\Big)\cos(k\psi)$	$-AJ_k(m)\Big(\cos\varphi-\cos(\delta\omega T+\varphi)\mathrm{sinc}(\delta\omega T)\Big)\sin(k\psi)$
$(I_{n,+}-I_{n,-})$	$-AJ_k(m)\Big(\sin\varphi-\sin(\delta\omega T+\varphi)\mathrm{sinc}(\delta\omega T)\Big)\sin(k\psi)$	$AJ_k(m)\Big(\cos\varphi-\cos(\delta\omega T+\varphi)\mathrm{sinc}(\delta\omega T)\Big)\cos(k\psi)$
$c_{n,+}$	$AJ_k(m)\Big(\cos\varphi+\cos(\delta\omega T+\varphi)\mathrm{sinc}(\delta\omega T)\Big)$	$AJ_k(m)\Big(\sin\varphi+\sin(\delta\omega T+\varphi)\mathrm{sinc}(\delta\omega T)\Big)$
$c_{n,-}$	$AJ_k(m)\Big(\sin\varphi-\sin(\delta\omega T+\varphi)\mathrm{sinc}(\delta\omega T)\Big)$	$AJ_k(m)\Big(\cos\varphi-\cos(\delta\omega T+\varphi)\mathrm{sinc}(\delta\omega T)\Big)$

Table S.2. Table of additional coefficients derived from Table S.1.

S.6 Algorithm performance in the case of non-white noise

S.6.1 Algorithm performance for a sudden 'step' and ring-down measurement

Figure S.5 shows the algorithm performance for two simulated cases where the end-mirror of an interferometer setup experiences a sudden step of 1 wavelength (1550nm). E.g. the position of the end mirror starts out constant but then receives an excitation

at some point and moves proportional to

$$L_1(t) = \begin{cases} 0.5\text{m} & \text{for } t < t_{\text{step}} \\ 0.5\text{m} + 1550\text{nm} \cdot e^{-1\text{Hz}(t - t_{\text{step}})} \cdot \cos(2\pi \cdot 10\text{Hz} \cdot (t - t_{\text{step}})) & \text{for } t >= t_{\text{step}} \end{cases} \tag{2}$$

$$L_2(t) = \begin{cases} 0.5\text{m} & \text{for } t < t_{\text{step}} \\ 0.5\text{m} + 1550\text{nm} \cdot e^{-10\text{Hz}(t - t_{\text{step}})} \cdot \cos(2\pi \cdot 100\text{Hz} \cdot (t - t_{\text{step}})) & \text{for } t >= t_{\text{step}} \end{cases} \tag{3}$$

with (2) corresponding to the upper plots and (3) corresponding to lower plots in Figure S.5. During the glitch, the algorithm does not operate correctly and returns erroneous values. In the case of a sudden excitation with a subsequent ring-down motion, the target can continues to move with significant speed making the readout also slightly less precise due to the motion as explained in more detail in section 5.

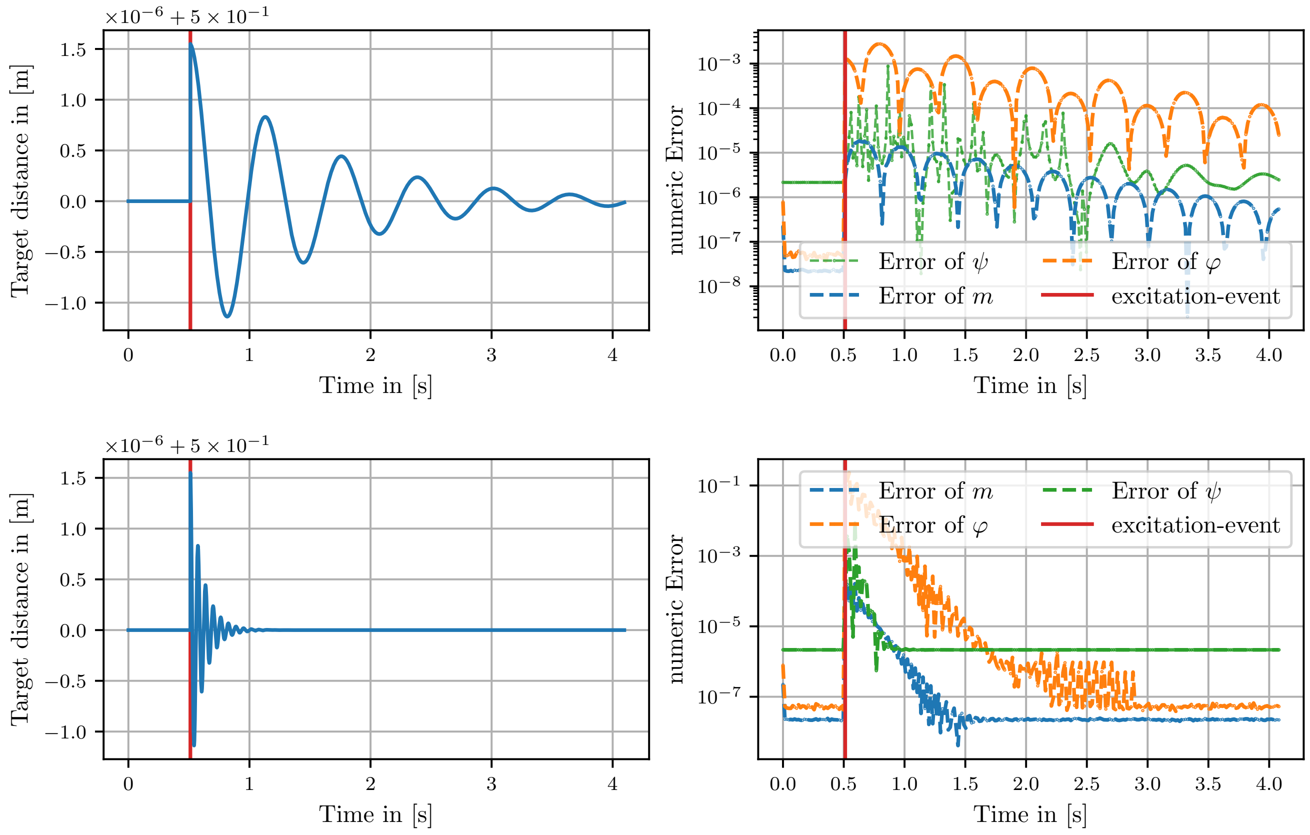

Figure S.5. Performance of the readout algorithm when the measured distance L suddenly changes and then rings down with an exponentially damped oscillation. The left plot show the simulated distance to the target and the right plot shows the parameter output of the algorithm during this period. The solid red line marks the moment of excitation. After the excitation event, the target still moves with significant speed during the ring-down movement, leading to a larger absolute error of the dynamic readout.

S.6.2 Algorithm performance in case of additive colored noise

In our simulations we find that the specific shape of any additive noise does not appear to influence the algorithm performance directly. As one would expect, it basically reduces the effective SNR at each (demodulated) harmonic frequency and thereby reduces the achievable phase readout noise level. For a known noise spectrum the weights can be adapted to optimise this SNR for any given case. Figure S.6 shows a DFMI signal with added $1/f^4$ noise and the algorithm results once with the regular weights defined in (15) and once with modified weights, where they include an additional f^2 factor. The modified weights yield on average a better result than the unmodified weights (which ignore the shape of the frequency dependant noise). This shows that, in case the shape of the non-white noise is known before the measurement, the precision of the algorithm can be improved by adapting the weights.

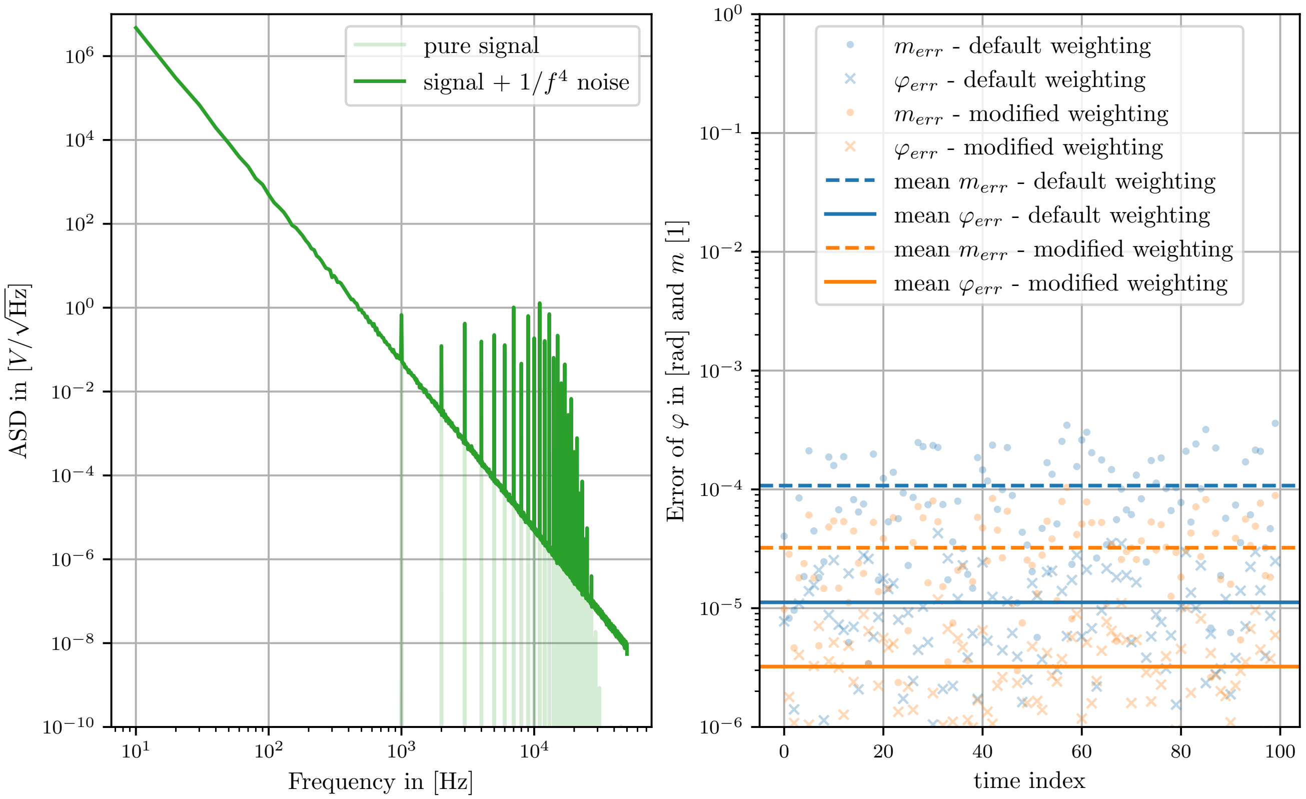

Figure S.6. Performance of the (static) readout algorithm in the presence of additive $1/f^4$-frequency noise. The left plot shows the average signal power spectrum with the added $1/f^4$ noise clearly visible. The right plot show the results of the algorithm run 100 times. The blue lines correspond to the algorithm using the default weighting of the harmonics as written in (15), and the yellow lines are the results when running the algorithm with the same data but modified weights with an additional f^2 factor. With the modified weight, and for this specific noise, the algorithm archives on average a higher precision compared to the default weights.

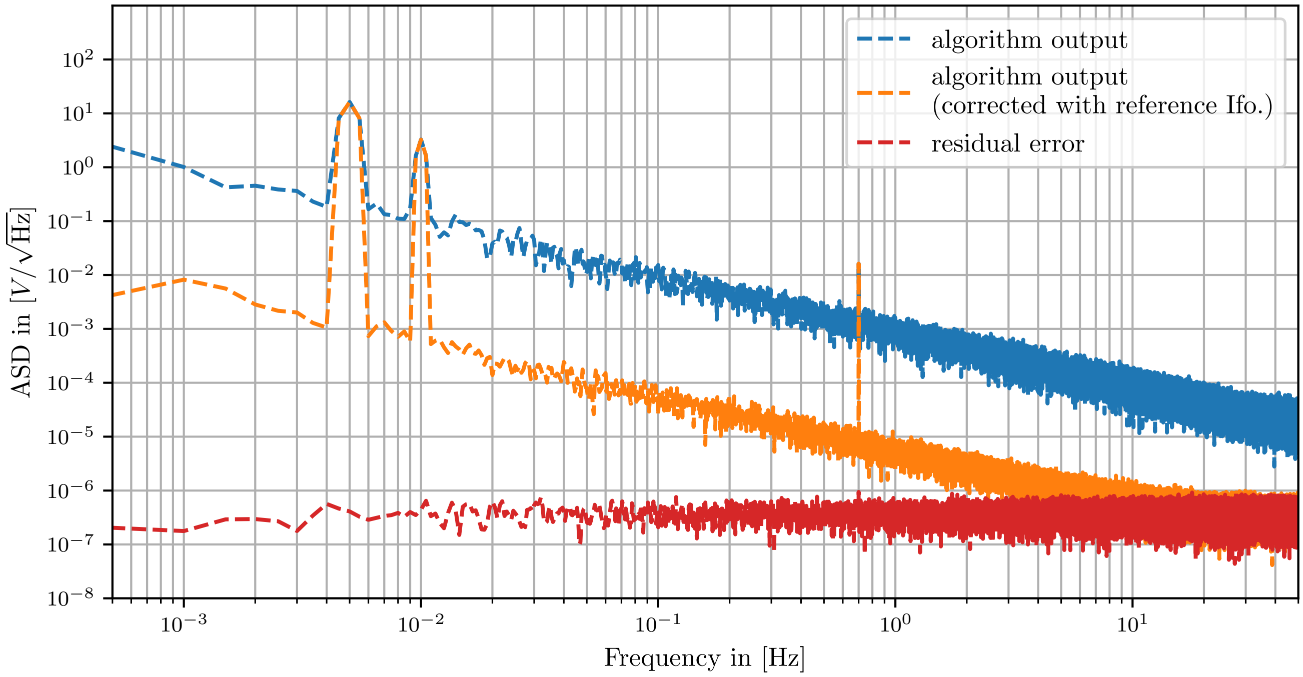

Figure S.7. Amplitude spectral density of the readout of a simulated DFM signal with $1/f$ laser frequency noise and three sinusoidal 'tones' and a reference interferometer signal. The plot uses the same signal data original used in[10] to generate Fig. 5, but the analytic algorithm is used to estimate the phase from this data.

S.6.3 Algorithm performance in case of $1/f$ laser-frequency noise

One of the primary noise sources in most interferometry techniques is laser frequency noise. Many high precision interferometers employ some kind of frequency stabilisation or frequency noise measurement scheme. For DFMI this is typically either a lock of the laser to, or simply the readout of, a stable reference interferometer without a moving mirror.

As part of our testing we recreated the simulation in[10] (there: Fig. 5, page 9) using the new algorithm. Simulated are two interferometers, one with a moving mirror and one with a stable arm and slightly different macroscopic arm-length. The stable interferometer measures effectively only the simulated laser frequency noise, which is then subtracted from the moving mirror measurement using the ratio of the two measured m values to scale the laser frequency noise. The results is then subtracted from the input phase of the simulation to reveal the precision and linearity of the algorithm. The result of this simulation is shown in Figure S.7. In the simulation the DFM setup includes $1/f$ colored frequency noise and multiple (non-linear) signals within the phase/position of the target. We used this as a verification that the readout algorithm preforms with similar precision as the previously used fit algorithms (which it does as seen from Figure S.7 (when compared with Fig. 5 in[10]).

Laser frequency noise can also couple directly at the modulation and harmonic frequencies, but here it is competing with the intentional laser frequency modulation, which is typically about 8 orders of magnitude larger. This effect is also contained in the above simulation, but shows no relevant effect.

S.7 Additional remarks

S.7.1 Absolute $|c_n|$ calculations, or bypassing the ψ calculation

It is also possible to calculate the absolute value $|c_n| := \sqrt{I_n^2 + Q_n^2}$ directly without the need to calculate ψ first. The following algorithm can in principle still work but will have several issues which can limit the precision of the results.

From the I_n and Q_n coefficient, the ψ parameter can be eliminated by calculating the absolute amplitude of each complex harmonic for later calculations of m and φ:

$$c_n := |\sqrt{I_n^2 + Q_n^2}| = \begin{cases} |A \cdot J_n(m) \cdot \cos\varphi| & \text{n even} \\ |A \cdot J_n(m) \cdot \sin\varphi| & \text{n odd.} \end{cases} \tag{4}$$

S.7.2 *Calculating the modulation index* m

Proceeding as before in section 3.4 and plugging the $|c_n|$ coefficients obtained from (4) into equation (10), the A and φ dependant factors cancel out again and only the $|J_n(m)|$ factors remain. E.g.

$$m_n \approx \sqrt{\frac{4n(n-1)(n+1)|c_n|}{2n|c_n|+(n+1)|c_{n-2}|+(n-1)|c_{n+2}|}} \tag{5}$$

$$= \sqrt{\frac{4n(n-1)(n+1)|J_n(m)|}{2n|J_n(m)|+(n+1)|J_{n-2}(m)|+(n-1)|J_{n+2}(m)|}} \tag{6}$$

This expression does however not equal equation (10) for m, as the inserted $|c_n|$ coefficients are absolute values and the 'sign' information of the included $|J_n(m)|$ factors is lost. This sign of the J_n coefficients can have a significant influence on the precision of the m estimation (e.g. $\mathcal{O}(10^0)$ compared to a desired sensitivities of $\mathcal{O}(10^{-8})$ for interferometry). For the case of $m=7$, $\varphi=\pi/4$ and $A=1$, the corresponding coefficient and the resulting m_n estimates from (11) are

$$\begin{aligned} c_2 &= -0.21313 \\ c_4 &= 0.1115 \\ c_6 &= 0.23985 \end{aligned} \overset{(11)}{\Longrightarrow} m_{\text{calc}} \approx 7 \tag{7}$$

but

$$\begin{aligned} c_2 &= +0.21313 \\ c_4 &= 0.1115 \\ c_6 &= 0.23985 \end{aligned} \overset{(11)}{\Longrightarrow} m_{\text{calc}} \approx 3.16 \tag{8}$$

Without the correct (minus) sign for (in this example) the c_2 coefficient, equation (11) (or (6 respectively)) yields wrong results. To deal with this ambiguity, we have one generic and one specific solution which can be used for sufficiently small changes in m over time.

The **generic solution** is to calculate the m_n coefficients for every possible combination of signs for the $|c_n|$. Every c_n has either a positive or negative sign which leads to $2^3 = 8$ possible combinations for every set of (J_{n-2}, J_n, J_{n+2}) but due to the symmetry of the equation (5), these 8 combinations reduce to only 4 different values for m_n. From the resulting set of values for m_n from all harmonics, we select the highest density region and average the m_n values in that region to get an estimate for m.

The **specific solution** (in case of continuous measurements) is to use the estimate of m from the previous calculation and 'correct' the signs of the c_n coefficients. E.g. we calculate $\text{sign}(J_n(m_{\text{previous}}))$ and use it as sign for the new c_n coefficients from the current measurement. For sufficiently small changes in m, $J_n(m_{\text{previous}})$ and $J_n(m_{\text{now}})$ share the same sign. With the correct signs, equation (5) yields the correct results for the m estimate.

Finally, we calculate the m estimate as before by performing a weighted average over the calculated m_n values as specified in section 3.7.

S.7.3 *Calculating* φ *from the* $|c_n|$

Eliminating the $|J_n(m)|$ factor from the $|c_n|$ factor similar to before now also yields (only) the absolute values of the φ quadratures, e.g.

$$\frac{|c_n|}{A|J_n(m)|} = \begin{cases} |\cos\varphi| & \text{n even} \\ |\sin\varphi| & \text{n odd.} \end{cases} \tag{9}$$

using the arctan again from the absolute $|\sin\varphi|/|\cos\varphi|$ coefficient effectively reduced the dynamic range to $\pi/4$ (a quarter of the wavelength).

S.7.4 *For large signal dynamics*

For dynamic signals ($\delta\omega$) the tracking of the individual tones and the calculation of the m parameter still works moderately well the same way as done before. The calculation of the φ parameter becomes however more unstable now. When proceeding as before one ends up with 4 absolute coefficients $|d_{+,\text{even}}|, |d_{+,\text{odd}}|, |d_{-,\text{even}}|, |d_{-,\text{odd}}|$. Before these coefficients were added together to expose the pure $\sin\varphi$ and $\cos\varphi$ quadratures. Without the correct signs this is no longer possible as e.g. $|d_{+,\text{odd}}| + |d_{-,\text{even}}|$ could either be $|\sin\varphi|$ or $|\sin(\varphi+\delta\omega T)|$ (with $|d_{+,\text{odd}}| - |d_{-,\text{even}}|$ being the other one).

We tested the algorithm by calculating 4 possible values for φ at this point via

$$\varphi_1 = \arctan\left(\frac{|d_{+,\text{odd}}| + |d_{-,\text{even}}|}{|d_{+,\text{even}}| + |d_{-,\text{odd}}|}\right)$$
$$\varphi_2 = \arctan\left(\frac{|d_{+,\text{odd}}| - |d_{-,\text{even}}|}{|d_{+,\text{even}}| + |d_{-,\text{odd}}|}\right)$$
$$\varphi_3 = \arctan\left(\frac{|d_{+,\text{odd}}| + |d_{-,\text{even}}|}{|d_{+,\text{even}}| - |d_{-,\text{odd}}|}\right)$$
$$\varphi_4 = \arctan\left(\frac{|d_{+,\text{odd}}| - |d_{-,\text{even}}|}{|d_{+,\text{even}}| - |d_{-,\text{odd}}|}\right) \tag{10}$$

and selecting the value closest to the previous measurement. We found however that in this case the algorithm often glitches and the φ calculation will generally be less precise compared to the case where the sign information of the c_n is kept and the ψ dependence is removed by hand as it is done in section 5.1.

S.7.5 Alternative way to calculate A and B

Since the individual harmonic amplitudes c_n (and the constant mean c_0) also scale with A and B, one can also calculate these parameters by eliminating the m and φ dependencies at the end of the algorithm and average again over the harmonics via:

$$A = \sqrt{\left(\frac{1}{N_{\text{even}}}\sum_{n\ \text{even}} \frac{c_n}{J_n(m)}\right)^2 + \left(\frac{1}{N_{\text{odd}}}\sum_{n\ \text{odd}} \frac{c_n}{J_n(m)}\right)^2} \tag{11}$$

$$B = \frac{c_0}{J_0(m)\cos\varphi} \tag{12}$$

with N_{even} and N_{odd} as number of used even and odd harmonics (to average over the values). We found however no benefit of using such more elaborate techniques as they have a similar error as the simpler approach in section 3.6.
